# An Integrated Lipidomics and Phenotype Study Reveals Protective Effect and Biochemical Mechanism of Traditionally Used *Alisma orientale* Juzepzuk in Chronic Kidney Disease

**DOI:** 10.3389/fphar.2018.00053

**Published:** 2018-02-08

**Authors:** Fang Dou, Hua Miao, Jing-Wen Wang, Lin Chen, Ming Wang, Hua Chen, Ai-Dong Wen, Ying-Yong Zhao

**Affiliations:** ^1^Department of Pharmacy, Xijing Hospital, Fourth Military Medical University, Xi'an, China; ^2^Key Laboratory of Resource Biology and Biotechnology in Western China, Ministry of Education, Northwest University, Xi'an, China

**Keywords:** Alisma orientale Juzepzuk, chronic kidney disease, inflammation, lipidomics, polyunsaturated fatty acid

## Abstract

*Alisma orientale* Juzepzuk (AO) is widely used for various diuretic and nephropathic treatments in traditional Chinese medicines (TCM). In a clinical setting, AO is used as both a lipid-lowering and tubular interstitial fibrosis agent. However, the mechanisms of AO for the treatment of renal interstitial fibrosis and abnormal lipid metabolism are not well-understood. In this study, pharmacological and UPLC-HDMS-based lipidomic approaches were employed to investigate the lipid-lowering and tubular interstitial fibrosis effect of AO on rats with adenine-induced chronic kidney disease (CKD). Rats with CKD showed increased serum levels of creatinine and urea, tubular damage, and tubular interstitial fibrosis. Moreover, multiple lipid species were identified in CKD rats. Among these lipids, polyunsaturated fatty acid, eicosapentaenoic acid, 8,9-epoxyeicosatrienoic acid, and docosahexaenoic acid levels were significantly decreased in CKD rats compared to control rats. In CKD rats, up-regulation of the NF-κB pathway may impair polyunsaturated fatty acid metabolism, causing renal fibrosis. In addition, CKD rats showed significantly decreased diglyceride levels and increased triglyceride levels compared to the control group. Pathway over-representation analysis demonstrated that 30 metabolic pathways were associated with lipid species. AO treatment suppressed up-regulation of inflammation, and partly restored the deregulation of polyunsaturated fatty acids and glycerolipids metabolism. Our results indicated that AO treatment attenuated renal fibrosis by down-regulating inflammation, and mitigating lipid metabolism in CKD rats. In conclusion, this study has identified the therapeutic lipid-lowering and anti-fibrosis effects of AO on CKD.

## Introduction

Epidemiological studies have suggested that in the general population the prevalence of chronic kidney disease (CKD) has increased over the past several decades (Lin et al., 2016). Worldwide, CKD poses a major burden to both affected patients and health care systems (Schefold et al., 2016). In patients with CKD, tubular atrophy and tubular interstitial fibrosis were the final common pathways that led to kidney disease progression and, ultimately, end-stage renal disease (Schefold et al., 2016). Traditional Chinese Medicine (TCM) has long been used in the clinic (Newman and Cragg, 2012; Mulders, 2013; Wang et al., 2017) and has been considered an alternative therapy for the treatment of various diseases, including the prevention and treatment of CKD (Zhao et al., 2012; Zhang Z. H. et al., 2015; Zhong et al., 2015; Zhang et al., 2016). In the general population, lipid metabolism disorders, which cause initiation and progression of atherosclerotic vascular changes, are major targets for preventive and therapeutic strategie**s** (Zhao, 2013; Chen D. Q. et al., 2014; Ridker, 2014). In CKD patients, lipid metabolism associated proteins that are involved in immune function and the acute phase response are modified (Weichhart et al., 2012; Zhao et al., 2015b,c). In TCM theory, nourishing qi, activating blood, and dissipating dampness are the main ways to treat CKD (Zhong et al., 2015), which requires increasing diuresis, reducing lipid metabolism disorders and preserving kidney function (Li and Wang, 2005; Chen D. Q. et al., 2017; Zhang et al., 2017). Previous studies have shown that *Alisma orientale* Juzepzuk (AO) has beneficial effects on dissipating dampness and promoting water metabolism in CKD (Chen H. et al., 2014; Feng et al., 2014; Chen L. et al., 2017).

In both Asia and Europe, the dried rhizome of AO (Zexie in Chinese), is widely used for its hyperlipidemic, anti-atherosclerotic, diuretic, nephropathic, and anti-diabetic properties (Miao et al., 2017). Its uses are described in numerous Chinese traditional medicine books (Table S1), such as Shen Nong's Herbal Classic (Shen Nong Ben Cao Jing), Treatise on Cold Febrile Induced Diseases (Shang Han Lun in Chinese), and Compendium of Materia Medica (Ben Cao Gang Mu in Chinese) (Li, 2007).

According to Chinese Materia Medica, AO was initially used as a medicine to treat edema and promote urinary excretion (Song, 1999). The rhizome of AO is the main medicinal component and has been used as an important ingredient in several TCM formulations for thousands of years, for example, in Liu Wei Di Huang Wan, Wu ling san, and Dang gui shao yao san, etc., These formulations contain AO, which has therapeutic effects on dysuria, cystitis, and diabetes and were mainly related to kidney and body fluid metabolism (Li, 2007). Several studies have indicated that AO may significantly decrease serum alanine transaminase, aspartate aminotransferase and relative liver weight in hyperlipidemic mice. Moreover, AO treatment may also reduce cholesterol and triglyceride levels in serum and liver when compared with a model group (Dan et al., 2011). Specifically, AO promotes urination and elimination of dampness, which are caused by the process of edema and urinary dysfunction. Previous studies have reported that the chemical constituents of AO mainly include triterpenes and sesquiterpenes (Table S2; Tian et al., 2014). However, the pharmacological activity of AO that is involved in attenuating renal interstitial fibrosis and modulating abnormal lipid metabolism is incompletely understood.

TCM possesses multi-component drug properties, which allow TCM to affect multiple targets (Martel et al., 2017). In agreement with the holistic thinking of TCM, lipidomics has shown great potential in evaluating both the therapeutic and toxic effects of TCM, as well as identifying the molecular mechanisms of action of TCM (Shi et al., 2016). Due to its enhanced analytic speed and sensitivity, as well as its high resolution of chromatographic peaks in complex mixtures, mass spectrometry-based lipidomics, ultra-performance liquid chromatography-quadrupole time-of-flight high-definition mass spectrometry (UPLC-QTOF/HDMS) has been widely used in metabolomics and lipidomics studies (Gao et al., 2016). Moreover, UPLC-QTOF/HDMS has been used to identify intact polar and neutral lipid molecular species (Zhao et al., 2015a). In this study, we combined the NF-κB/Nfr2 and TGF-β/Smad signaling pathways and UPLC-HDMS-based serum to investigate the lipid profiles and potential lipid biomarkers in adenine-induced CKD rats that were treated with an ethyl acetate fraction of AO. In this study, we explain the pathological changes of CKD, in addition to the lipid-lowering and anti-fibrotic mechanisms of action of AO.

## Materials and methods

### Chemicals and reagents

Adenine and formic acid were purchased from Sigma Chemical Co. (St. Louis, MO, USA). Creatinine (batch No.: 100877-200901, Purity 99.8%) was obtained from the National Institutes for Food and Drug Control (Beijing, China). LC-grade methanol and acetonitrile were purchased from the Baker Company. Ultra-high purity water was prepared using a Milli-Q water purification system. Other chemicals were of analytical grade and purity was above 99.5%.

### Animals and the preparation of ethyl acetate

Sprague-Dawley rats (male, aged 6 weeks, 180–200 g) were obtained from the Experimental Animal Center of the Fourth Military Medical University (Xi'an, China). Rats were maintained at a constant humidity (~60%) and temperature (~23°C) with a light/dark cycle of 12 h. Experimental studies were approved by the Ethics Committee for Animal Experimentation of the Northwest University. The ethical approval reference number of the study is SYXK2010-004.

In January 2016, AO was collected from Fujian Province and was identified by Prof. M. F. Fang (School of Life Science, Northwest University, Xi'an, Shaanxi, China). A voucher specimen (Z150319) was deposited at the School of Life Science, Northwest University, Xi'an, Shaanxi. Crushed AO (2.5 kg) was extracted three times with 95% ethanol at room temperature for 5 days each time. The extraction rate of the ethanol extract of AO was 6.44%. The filtrates were concentrated under reduced pressure to give a crude reddish-brown extract, which was then dissolved in H_2_O. The suspension was successively fractionated three times with petroleum ether, ethyl acetate, and n-BuOH, and concentrated under reduced pressure to yield four fractions. Previous studies have shown that the ethyl acetate fraction of AO contained the active compound (Feng et al., 2014). The ethyl acetate fraction of AO was orally administered to rats.

### Chronic kidney disease model and drug administration

Male rats underwent an adaptation period of several days, during which they were fed commercial feed. Rats weighing 180 g to 200 g were randomly divided into three groups: (1) healthy control group (*n* = 8), (2) CKD model group (*n* = 8), and (3) AO-treated group with CKD (*n* = 8). Groups 2 and 3 were given 200 mg/kg body weight of adenine dissolved in 1% (w/v) gum acacia solution, administered by oral gavage once a day for three consecutive weeks (Zhao et al., 2013). Similarly, group 1 was given an equal volume of gum acacia solution. Group 3 received, 3 h after adenine gastric gavage, the ethyl acetate extract (130 mg/kg) by gastric irrigation during the 6-week study period.

### Sample collection

Three weeks after the start of treatment, rats were anesthetized using 10% urethane, and blood samples were obtained by the carotid artery cannula. Blood was centrifuged at 3,000 rpm for 10 min, after which the serum was collected and stored at −80°C. After blood was collected, kidneys were harvested immediately and were washed with saline.

### Determination of the physiological and biochemical parameters

At the end of the experiment, rats were housed individually for 24 h in metabolic cages for urinary collection. Body weights were recorded after the 24 h time-period. Upon collection of their urine samples, rats were allowed free access to water. Urine samples were stored at −80°C prior to analysis, and kidneys were weighed to calculate organ indexes (organ index = organ weight/body weight ×100%). Serum levels of creatinine, urea, cholesterol, triglyceride, low density lipoprotein-cholesterol (LDL-C), and high density lipoprotein-cholesterol (HDL-C) were measured using an Olympus AU640 automatic analyzer.

### Histopathology and immunohistochemistry

A portion of each fresh kidney was immersed in 10% neutral, buffered formaldehyde solution, then dehydrated, embedded in paraffin, cut in 5 μm sections, and stained with Hematoxylin and Eosin (H&E) and Masson staining for histopathological examination. Immunohistochemical staining was performed using the published procedure (Chan et al., 2017). The antibodies used were directed against the following: nuclear factor kappa B p65 (NF-κB p65), cyclooxygenase-2 (COX-2), Monocyte Chemoattractant Protein-1 (MCP-1), inducible nitric oxide synthase (iNOS), tumor necrosis factor-alpha (TNF-α), Nrf2, heme oxygenase-1 (HO-1), transforming growth factor-β1 (TGF-β1), transforming growth factor-β receptor II (TGF-β RII), Smad2, Smad3, Smad4, Smad7, fibronectin, alpha-smooth muscle actin (a-SMA), plasminogen activator inhibitor-1 (PAI-1), a-SMA, collagen I, and glyceraldehyde-3-phosphate dehydrogenase (GAPDH). Antibodies were purchased from Abcam (Cambridge, United Kingdom), Cell Signaling Technology (Danvers, MA), or Santa Cruz Biotechnology, Inc., (Santa Cruz, CA). Image analysis was performed by using Image-Pro Plus 6.0 software.

### Western blot analysis

Proteins were extracted using radioimmunoprecipitation assay buffer, which contained a cocktail of proteinase inhibitors (Thermo Fisher Scientific Inc., Rockford, IL), and were quantified with a Bio-Rad protein assay. Equal amounts of protein were separated on 10% SDS-polyacrylamide gels in a Tris/HCl buffer system, transferred onto nitrocellulose membranes, and blotted according to standard procedures. Nonspecific proteins were blocked by incubating the membrane with 5% nonfat dried milk in TBS-T for 1 h at room temperature with agitation. Membranes were then incubated overnight at 4°C with the primary antibodies directed against PAI-1, a-SMA, collagen I, or GAPDH. Subsequently, membranes were washed and then incubated with goat anti-rabbit IgG or goat anti-mouse IgG secondary antibodies (Abcam, Cambridge, MA) for 1 h at room temperature (Pelletier et al., 2017). Specific bands indicating target proteins were analyzed using ImageJ software.

### Sample preparation and UPLC-HDMS analysis

For the lipid profiling of blood samples, UPLC-HDMS was used. The extraction of total lipids by the Ostro 96-well plate system was performed as a single-step in-well extraction. Samples for lipidomics were prepared as previously described (Chen H. et al., 2017).

UPLC-HDMS was performed on a Waters Acquity Ultra Performance LC system, equipped with a Waters Xevo G2 QTof MS. UPLC analysis was performed using a HSS T3 column. The samples were characterized following our previously published methods with minor modifications (Chen H. et al., 2017). All acquisitions were operated using Waters MassLynx v4.1 software.

### Pattern recognition analysis and data processing

Precision and reproducibility were verified by previously described methods (Zhao et al., 2013). In brief, raw data were imported to Markerlynx XS (Waters Corporation, MA, USA) for peak detection and alignment. Data were normalized to the summed total ion intensity per chromatogram and the resultant data matrices were loaded into EZinfo 2.0 software (Umetrics Corporation, Sweden) for principal component analysis (PCA) (Zhao et al., 2013). Partial least-squares discriminant analysis (PLS-DA) was performed and potentially important variables were selected according to the variable importance in the projection (VIP) values, which reflected the contribution of each variable in the three groups. Metabolite peaks were assigned by MSE analysis or interpreted with available biochemical databases: HMDB (http://www.hmdb.ca/), KEGG (http://www.genome.jp/kegg/pathway.html) and Chemspider (http://www.chemspider.com) (Zhao et al., 2013). From the identified lipid species, heatmap, fold changes (CKD/control, CKD+AO/CKD, or CKD+AO/control), and receiver-operating characteristic (ROC) curves were analyzed by Metaboanalyst 3.0 and Medcalc 12.7. The one-way analysis of variance (ANOVA) or Mann–Whitney U test was used to calculate statistical significance with SPSS 22.0 software. The false discovery rate (FDR) correction was calculated to reduce the risk of a false positive value by the adjusted *p* < 0.05 based on the Benjamini Hochberg method. Values of *p* < 0.05 were considered significant.

### Construction of a metabolic network

In order to construct a metabolic network of identified lipids, we integrated interaction databases: Reactome, Database for Annotation, Visualization and Integrated Discovery (DAVID), Kyoto Encyclopedia of Genes and Genomes (KEGG), Biomolecular Interaction Network Database (BIND), Human Protein Reference Database (HPRD), BioGrid, Database of Interacting Proteins (DIP), Metabolite Set Enrichment Analysis (MSEA), and Quantitative Enrichment Analysis (QEA) (Xenarios et al., 2002; Bader et al., 2003; Huang da et al., 2009; Goel et al., 2012; Xu et al., 2017). These databases contained the most comprehensive publicly available repository of genes, proteins, and interaction of complexes for Homo sapiens. In these integrative databases, sets of lipid species from metabolite-related networks were mapped to their related molecular pathways and networks.

## Results

### Physiological and biochemical parameters

**Figure 1:**
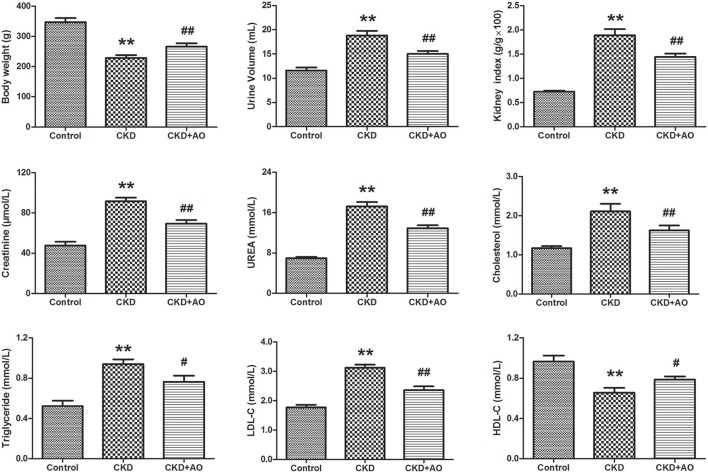
Physiological and biochemical parameters. Body weight, urine volume, kidney weight index, serum creatinine, urea, cholesterol, triglyceride, LDL-C, and HDL-C parameters in the control, adenine-induced CKD and CKD+AO groups. All values presented as mean ± SD (n = 8 for each group). All *p*-values were calculated by two-tailed Student's *t*-test with 95% confidence interval. ^**^p < 0.01 compared with control group, ^#^*p* < 0.05, ^##^*p* < 0.01 compared with CKD group.

In Figure 1, body weight, urine volume, kidney weight index, creatinine, urea, cholesterol, triglyceride, LDL-C, and HDL-C are given for the three groups. Our data indicated that body weight, urinary volume, and kidney weight index were significantly decreased in the CKD group compared to the control group (*p* < 0.01). However, body weight, urinary volume, and kidney weight index were markedly increased in the AO-treated group compared to the CKD group. In addition, levels of serum creatinine, urea, cholesterol, triglyceride, and LDL-C were significantly increased in the CKD group compared to the control group (*p* < 0.01). Moreover, HDL-C levels were significantly decreased in the CKD group compared to the control group. These biochemical parameters were improved after treatment with AO.

### AO mitigates inflammatory responses in adenine-induced CKD rats

**Figure 2:**
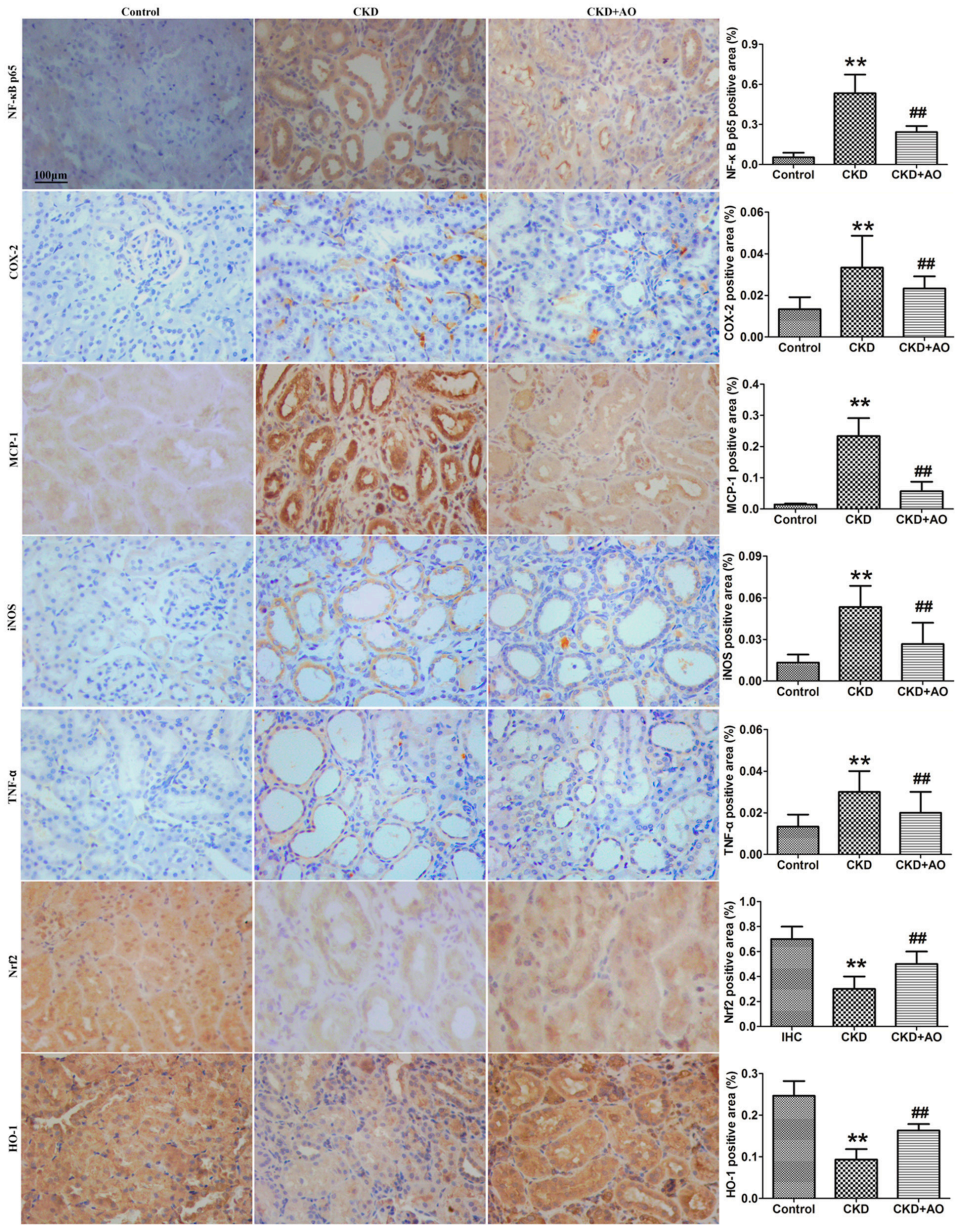
Immunohistochemical findings with anti-NF-κB p65, COX-2, MCP-1, iNOS, TNF-α, Nrf2 and HO-1 antibodies in the control, adenine-induced CKD and CKD+AO groups of rat kidneys. ^**^p < 0.01 compared with control group; ^##^*p* < 0.01 compared with CKD group. The data are presented as the mean ± SD (*n* = 8 in each group).

Compared to control rats, adenine-induced CKD rats showed significant up-regulation in the nuclear translocation of p65 protein expression, indicating the activation of NF-κB signaling. In adenine-induced CKD rats, NF-κB activation was accompanied by a significant up-regulation of inflammatory proteins, such as COX-2, MCP-1, iNOS, and TNF-α, and down-regulation of the anti-oxidant system, including Nrf2, and HO-1. Compared with CKD rats, up-regulation of NF-κB p65, COX-2, MCP-1, iNOS, and TNF-α protein expression, and down-regulation of Nrf2 and HO-1 protein expression was attenuated by AO treatment (Figure 2). Thus, the possible mechanisms underlying the nephropathic effect of AO may involve the TGF-β1/Smad signaling pathway.

### AO blocks TGF-β1/Smad signaling in the adenine-induced chronic kidney disease rats

**Figure 3:**
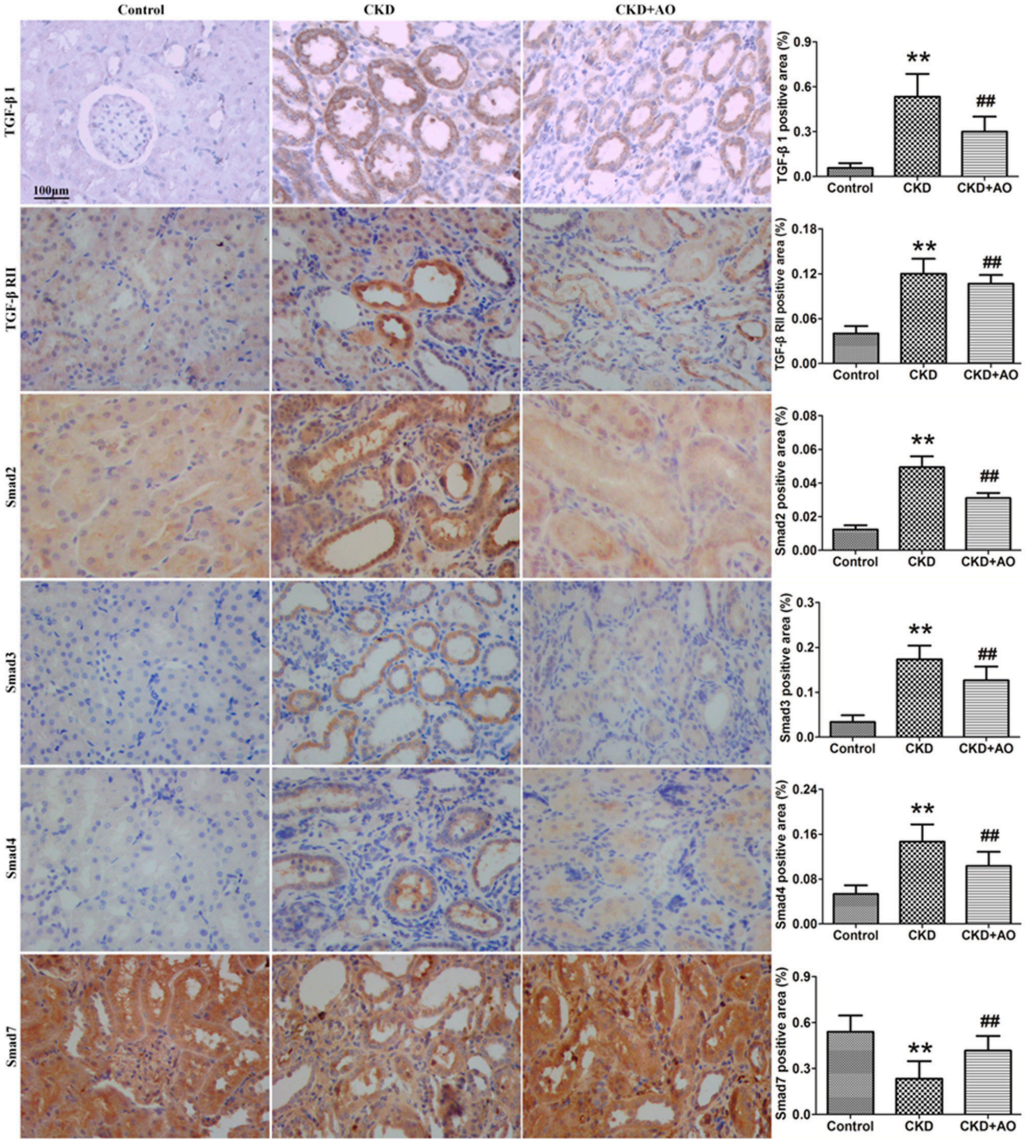
Immunohistochemical findings with anti-TGF-β1, TGF-β RII, Smad2, Smad3, Smad4, and Smad7 antibodies in the control, adenine-induced CKD and CKD+AO groups of rat kidneys. ^**^p < 0.01 compared with control group; ^##^*p* < 0.01 compared with CKD group. The data are presented as the mean ± SD (*n* = 8 in each group).

Activation of the TGF-β1/Smad signaling pathway contributes to renal interstitial fibrosis (Ma et al., 2010). Compared to control rats, protein expression of TGF-β1 and TGF-β RII were significantly up-regulated in adenine-induced CKD rats (Figure 3). This up-regulation was accompanied by significant up-regulation of Smad2, Smad3, Smad4, and Smad7 protein expression in adenine-induced CKD rats, indicating activation of the TGF-β1/Smad signaling pathway. Compared to CKD rats, activation of the TGF-β1/Smad signaling pathway was attenuated in AO-treated rats (Figure 3). Therefore, the possible mechanisms underlying the nephropathic effect of AO may involve the NF-κB/Nrf2 signaling pathway.

### AO alleviates renal interstitial injury and fibrosis in adenine-induced chronic kidney disease rats

**Figure 4:**
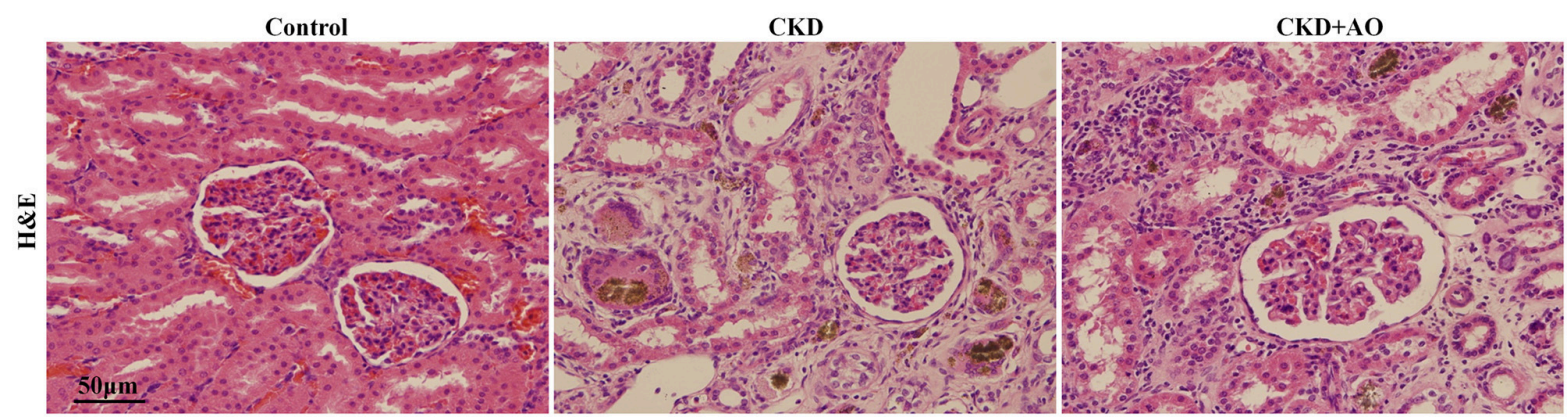
H&E staining of kidney tissue. Representative images of H&E stained kidney sections from control, adenine-induced CKD and CKD+AO groups.

**Figure 5:**
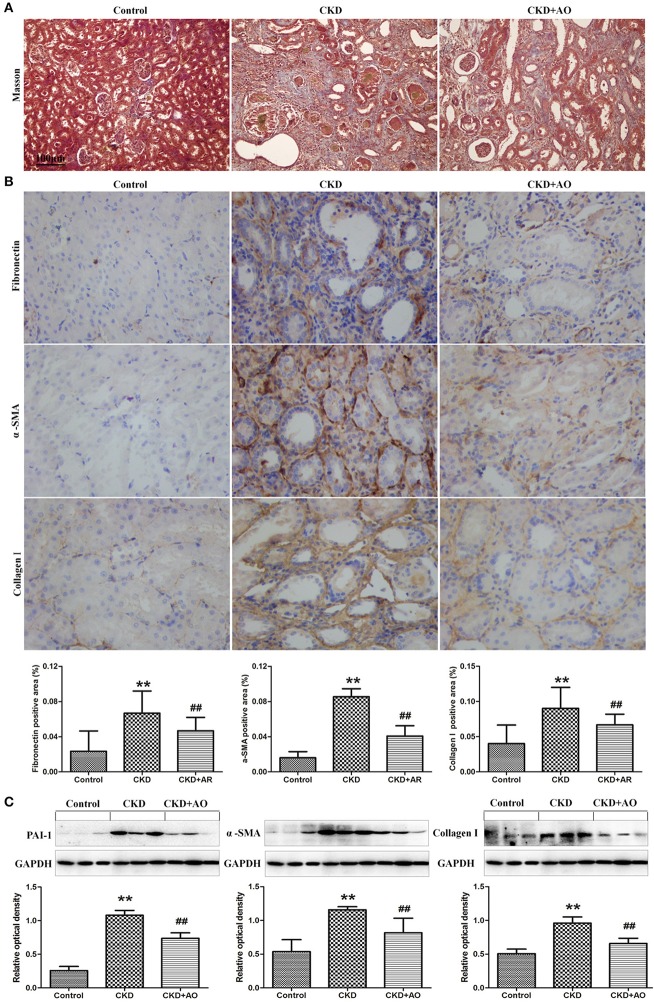
Masson staining and Western blot analysis of kidney tissue. **(A)** Representative images of Masson stained kidney sections from control, adenine-induced CKD and CKD+AO groups. **(B)** Immunohistochemical findings with anti-Fibronectin, α-SMA and Collagen I antibodies in the control, adenine-induced CKD and CKD+AO groups of rat kidneys. **(C)** Protein expression of PAI-1, α-SMA, and collagen I in the renal tissues of control, adenine-induced CKD and CKD+AO groups. ^**^p < 0.01 compared with control group; ^##^*p* < 0.01 compared with CKD group. The data are presented as the mean ± SD (*n* = 8 in each group).

Figures 4, 5 demonstrate the H&E and Masson immunohistochemical staining results, and Western Blot analysis of kidney tissue derived from adenine-induced rats. The formation of foreign body granuloma in the renal tubules and renal interstitial fibrosis, as well as a significant degree of renal fibrosis was observed. Histological analysis demonstrated that in adenine-induced rats, typical pathological features of CKD were observed (Figures 4, 5). In contrast, these pathological abnormalities were alleviated in AO-treated rats. Adenine administration resulted in a significant increased protein expression of fibronectin, collagen I, PAI-1 and α-SMA, whereas AO administration reduced the expression of these molecules compared to CKD rats (Figure 5). In addition, in adenine-induced CKD rats, significant up-regulation of pro-fibrotic proteins was accompanied by activation of inflammatory and TGF-β1/Smad pathways. In conclusion, AO attenuated NF-κB/Nrf2, TGF-β1/Smad, and pro-fibrotic pathways in adenine-induced CKD rats.

### Selection and identification of important differential lipid species

For all analyses, reproducibility was confirmed by performing six replicated determinations of each plasma sample. For the method validation, eight ions, including 2.40_320.3081, 7.81_742.6677, 6.30_647.4589, 4.46_1194.8158, 5.02_662.4451, 8.03_784.7189, 5.02_722.5260, and 8.34_700.6142, were selected. The relative standard deviation values of the retention time and peak area were <0.73 and 2.95%, respectively. These results indicated sufficient reproducibility of these analyses.

**Figure 6:**
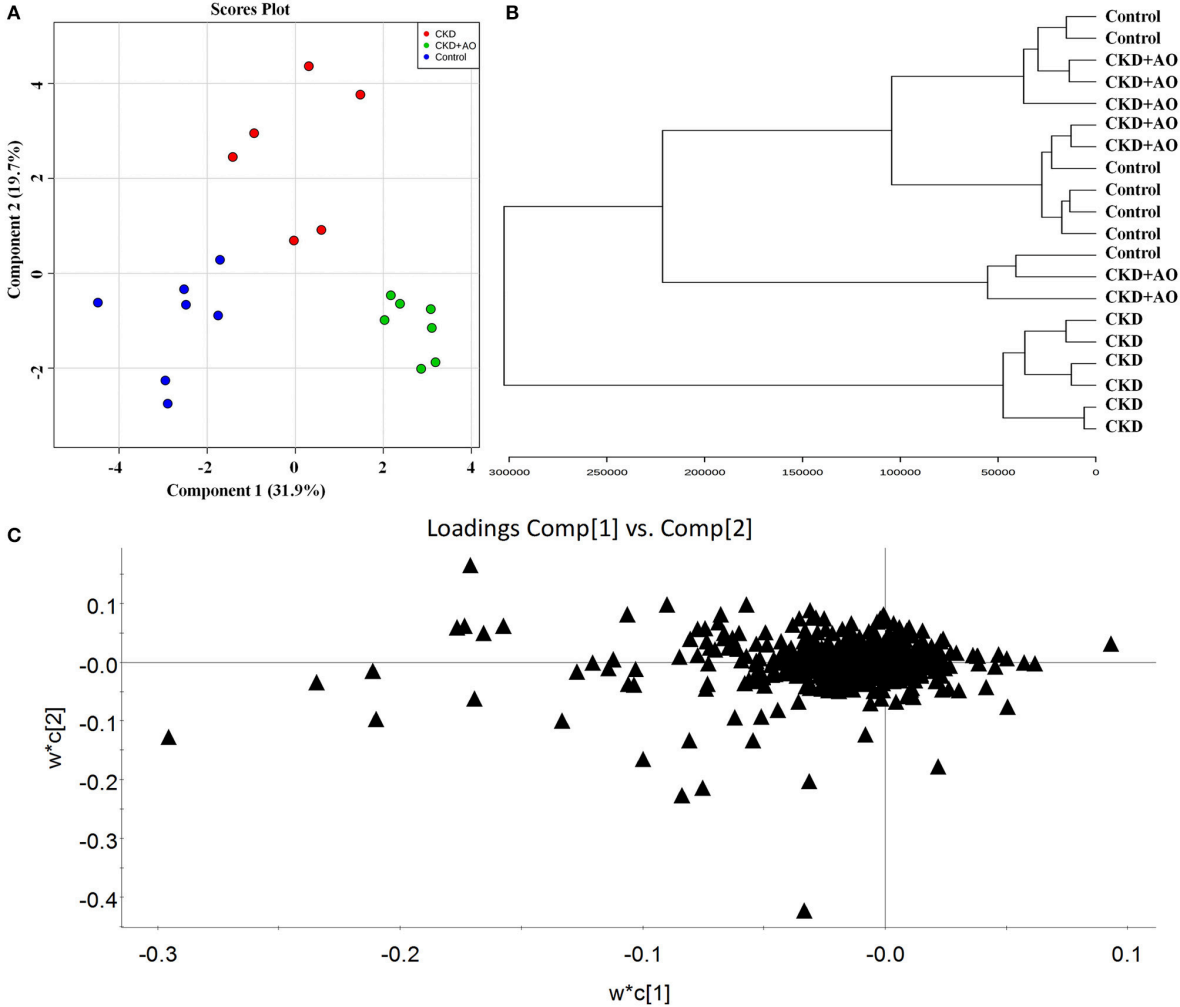
Lipid profiling and multivariate statistical analysis. **(A)** PLS-DA model for control, adenine-induced CKD and CKD+AO groups. **(B)** Clustering analysis of control, adenine-induced CKD, and CKD+AO groups. **(C)** Loadings plot of PLS-DA in positive and negative ion mode from control, adenine-induced CKD, and CKD+AO groups.

In order to evaluate the ability of AO to ameliorate the lipid metabolic profile in adenine-induced CKD rats, the two-predictive component PLS-DA model [R2X(cum) = 0.961, Q2(cum) = 0.751] was used. In both the positive and negative ion modes, this model showed a satisfactory separation capacity by 384 variables among the three groups (Figure 6A). As shown in the clustering analysis, adenine-induced CKD rats can be separated from control and AO-treated rats, however AO-treated rats cannot be separated from the control rats (Figure 6B). Therefore, these data demonstrated the efficacy of AO in improving treatment of CKD rats.

**Figure 7:**
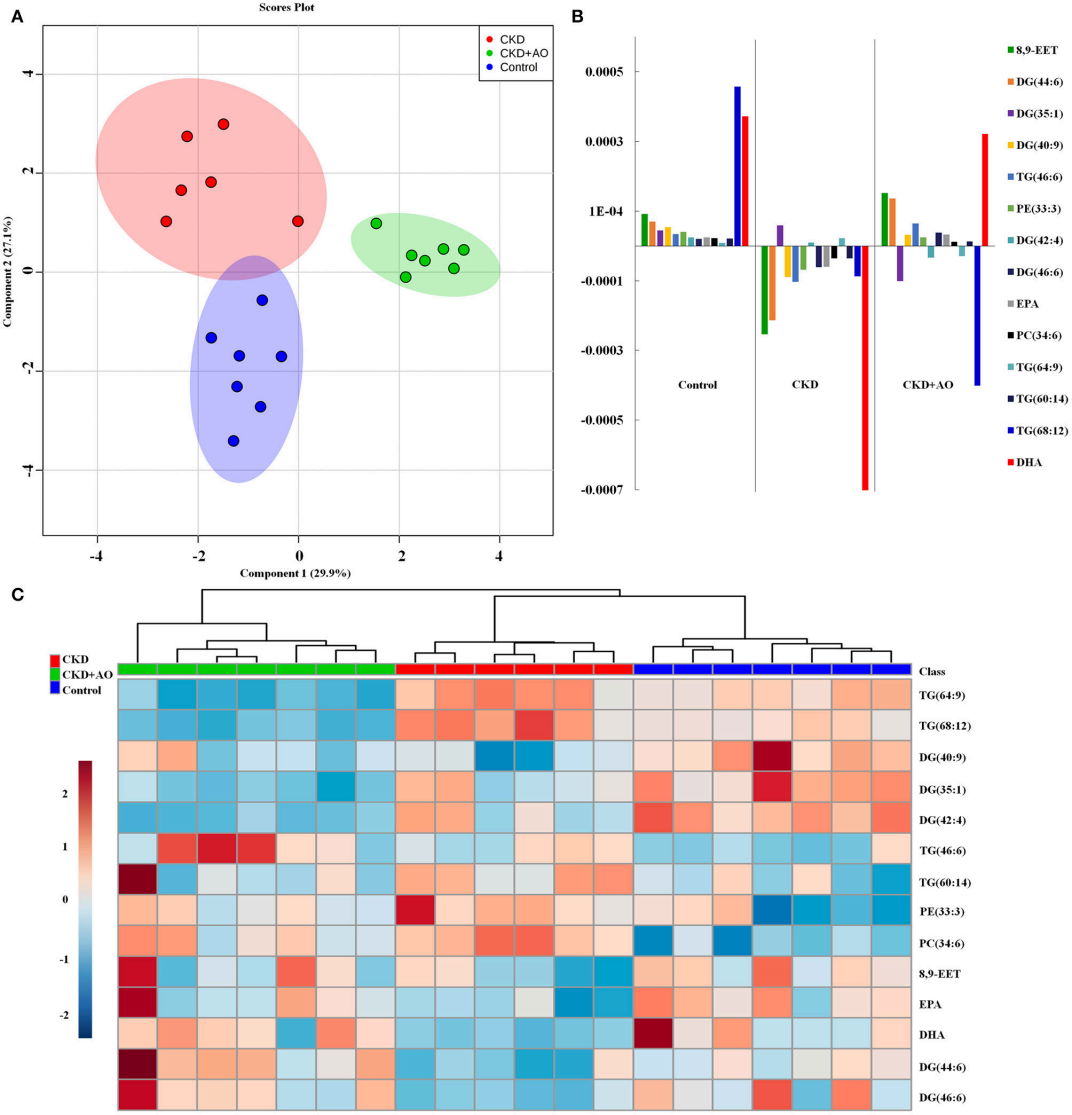
Lipid metabolite identification and correlation analysis. **(A)** PCA of two components of 14 lipid species from control, adenine-induced CKD, and CKD+AO groups. **(B)** Correlation coefficient analysis of 14 lipid species among control, adenine-induced CKD, and CKD+AO groups. **(C)** Heatmap of 14 lipid species among control, adenine-induced CKD and CKD+AO groups. Red and blue indicate increased and decreased levels, respectively.

To identify altered lipid species, variables were selected using VIP values based on the PLS-DA model: 383 variables in the positive ion mode and 58 variables in the negative ion mode had a VIP value of >1.0 (Figure 6C). Based on the one-way ANOVA analysis and Mann–Whitney U test (*p* < 0.05), 100 variables in the positive ion mode and 23 variables in the negative ion mode were selected for further analysis. After combining the FDR analysis (*p* < 0.05) and the ROC curves (AUC > 0.80), a total of 20 variables were selected for the identification of lipid metabolites in both the positive and negative ion modes. Xenobiotics and different fragment ions from the same lipid species were excluded, and 14 differential lipid species were identified (Table 1). These lipids included five diglycerides, four triglycerides, three fatty acids, one phosphatidylcholine, and one phosphatidylethanolamine. In AO-treated rats, changes in eight lipid species were completely restored when compared to CKD rats. In addition, five lipid species were restored to normal levels in the AO-treated group compared to the control group. The PCA score plot of the 14 lipid species in the AO-treated group was located between those of the CKD and control rats (Figure 7A), which was consistent with the results of the heatmap analysis (Figure 7C). Moreover, Figure 7B indicates the correlation coefficient analysis among lipid species and their corresponding groups. Lipid species situated in the upper panel positively correlated with the corresponding group, whereas those situated in the opposite panel negatively correlated with the corresponding group. The lipid species 8,9-epoxyeicosatrienoic acid (8,9-EET), DG (44:6), DG (40:9), TG (46:6), PE (33:3), DG (46:6), eicosapentaenoic acid (EPA), PC (34:6), TG (60:14), and docosahexaenoic acid (DHA) were positively correlated with control rats, whereas all other lipid species were negatively correlated with control rats. The correlation coefficient was significantly altered in adenine-induced CKD rats, indicating that the significant abnormalities of the lipid metabolic profile were caused by adenine. In AO-treated rats, nine lipid species showed similar altered tendencies observed in control rats. The results demonstrated that AO ameliorated abnormal lipid metabolism in CKD-afflicted rats using adenine, which coincides with our biochemical results.

**Table 1:** Identified plasma lipids, fold changes (FC) and *p*-values among CTL, CKD, and CKD + AO groups.

**Metabolite**		**CKD vs. Control**				**CKD + AO vs. CKD**				**CKD + AO vs. Control**			
	**VIP^a^**	**FC^b^**	***p*-value^c^**	***p*-value^d^**	**FDR^e^**	**FC^b^**	***p*-value^c^**	***p*-value^d^**	**FDR^e^**	**FC^b^**	***p*-value^c^**	***p*-value^d^**	**FDR^e^**
8,9-EET	6.9	0.76	2.94 × 10^−02^	3.50 × 10^−02^	3.20 × 10^−02^	1.26	2.05 × 10^−01^	3.66 × 10^−01^	3.78 × 10^−04^	0.96	7.63 × 10^−01^	5.35 × 10^−01^	1.40 × 10^−05^
DG(44:6)	4.7	0.51	1.93 × 10^−02^	3.50 × 10^−02^	2.61 × 10^−02^	3.05	1.80 × 10^−02^	1.4 × 10^−02^	3.36 × 10^−02^	1.57	6.14 × 10^−01^	7.10 × 10^−01^	7.00 × 10^−06^
DG(35:1)	4.2	0.73	3.70 × 10^−02^	2.21 × 10^−02^	3.78 × 10^−02^	0.60	4.80 × 10^−03^	4.70 × 10^−03^	2.49 × 10^−02^	0.44	3.70 × 10^−05^	5.83 × 10^−04^	1.73 × 10^−04^
DG(40:9)	2.4	0.81	4.92 × 10^−03^	1.20 × 10^−03^	2.05 × 10^−02^	1.07	2.72 × 10^−01^	7.31 × 10^−01^	2.23 × 10^−02^	0.87	2.04 × 10^−02^	2.62 × 10^−02^	5.72 × 10^−02^
TG(46:6)	2.3	1.47	2.27 × 10^−02^	2.21 × 10^−02^	2.83 × 10^−02^	1.37	7.62 × 10^−03^	2.30 × 10^−03^	2.48 × 10^−02^	2.01	7.43 × 10^−01^	9.02 × 10^−01^	9.42 × 10^−02^
PE(33:3)	2.1	1.35	2.50 × 10^−02^	1.40 × 10^−02^	3.01 × 10^−02^	0.88	3.90 × 10^−01^	6.28 × 10^−01^	3.60 × 10^−02^	1.18	4.03 × 10^−02^	3.79 × 10^−02^	8.38 × 10^−02^
DG(42:4)	1.6	0.61	1.88 × 10^−02^	3.50 × 10^−02^	2.58 × 10^−02^	0.33	5.34 × 10^−03^	4.70 × 10^−03^	9.08 × 10^−02^	0.20	1.00 × 10^−06^	5.83 × 10^−04^	2.12 × 10^−01^
DG(46:6)	1.3	0.28	2.04 × 10^−02^	2.21 × 10^−02^	2.62 × 10^−02^	4.04	7.96 × 10^−03^	4.70 × 10^−03^	9.33 × 10^−02^	1.11	7.33 × 10^−01^	9.02 × 10^−01^	5.24 × 10^−01^
EPA	1.3	0.57	6.94 × 10^−03^	1.40 × 10^−02^	1.98 × 10^−02^	1.65	6.00 × 10^−02^	5.13 × 10^−02^	2.87 × 10^−01^	0.94	7.50 × 10^−01^	5.35 × 10^−01^	8.60 × 10^−01^
PC(34:6)	1.1	1.26	2.94 × 10^−02^	1.40 × 10^−02^	3.27 × 10^−02^	0.93	4.36 × 10^−01^	8.36 × 10^−01^	4.56 × 10^−01^	1.18	4.19 × 10^−02^	7.28 × 10^−02^	8.67 × 10^−01^
TG(64:9)	1.1	1.22	1.51 × 10^−02^	2.21 × 10^−02^	2.29 × 10^−02^	0.23	2.70 × 10^−05^	1.20 × 10^−03^	4.70 × 10^−01^	0.27	1.00 × 10^−06^	5.83 × 10^−04^	8.07 × 10^−01^
TG(60:14)	1.1	1.32	1.10 × 10^−02^	1.40 × 10^−02^	2.16 × 10^−02^	0.86	4.81 × 10^−01^	6.28 × 10^−01^	4.81 × 10^−01^	1.13	1.21 × 10^−01^	9.73 × 10^−02^	7.63 × 10^−01^
TG(68:12)	1.3	1.39	1.47 × 10^−02^	8.00 × 10^−03^	1.88 × 10^−02^	0.27	3.82 × 10^−02^	9.31 × 10^−02^	5.73 × 10^−02^	0.37	7.10 × 10^−05^	6.66 × 10^−04^	7.28 × 10^−02^
DHA	1.0	0.41	3.08 × 10^−02^	2.93 × 10^−02^	3.08 × 10^−02^	2.07	3.46 × 10^−02^	4.11 × 10^−02^	2.15 × 10^−01^	0.85	7.47 × 10^−01^	9.50 × 10^−01^	5.01 × 10^−01^

a *VIP values were obtained from the CKD model*.

b *Fold change (FC) was calculated based on a binary logarithm for CKD vs. Control and CKD + AO vs. CKD. FC with a value greater than zero indicates a higher intensity of the plasma metabolite between CKD vs. Control and between CKD + AO vs. CKD, while a FC value less than zero indicates a lower intensity of the plasma metabolite between CKD vs. Control and between CKD+AO vs. CKD*.

c *p-values are calculated by one-way ANOVA*.

d *p-values are calculated by nonparametric Mann-Whitney U-test*.

e *The false discovery rate (FDR) was obtained from the adjusted p-value of the FDR correction using Benjamini–Hochberg method*.

### ROC curve analysis

**Figure 8:**
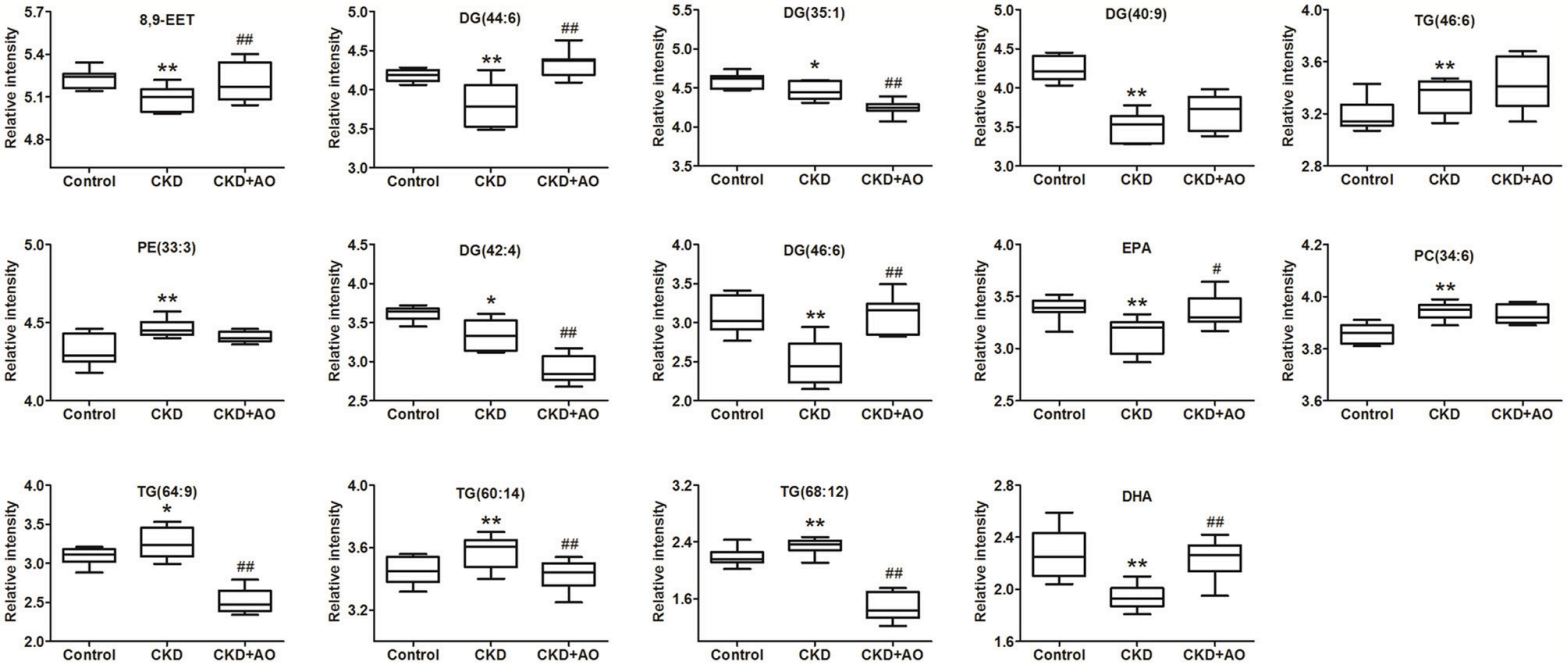
Relative intensity analysis of 14 lipid species. Box plots showing significant changes in the levels of 14 lipid species among the control, adenine-induced CKD and CKD+AO groups. The statistical significance between the two groups is marked. ^*^p < 0.05, ^**^p < 0.01 significant difference compared with control group; ^#^*p* < 0.05, ^*##*^*p* < 0.01 significant difference compared with adenine-induced CKD group. Y-axis: normalized relative intensity.

PLS-DA-based ROC curves were performed to further identify potential lipid biomarkers that would indicate the therapeutic effects of AO on CKD. The area under the curve (AUC), 95% confidence interval (95%CI), sensitivities, and specificities of 14 lipid species are shown in Figure S5. Seven lipid species, including DG (44:6), DG (35:1), TG (46:6), DG (42:4), DG (46:6), TG (64:9), and DHA had a high AUC value (>0.80), sensitivity (>80%), and specificity (>80%). These lipid species included four diglycerides, two triglycerides, and one fatty acid and could be considered as potential lipid biomarkers of the therapeutic effects of AO on CKD. Figure 8 demonstrates the levels of the 14 lipid biomarkers among the three groups. In conclusion, these results indicate that diglycerides, triglycerides, and polyunsaturated fatty acids can serve as potential biomarkers in adenine-induced CKD rats, and that AO mitigates the abnormal lipid metabolism, and exhibits anti-fibrotic effects.

### Perturbed metabolic network in CKD and its response to AO therapy

**Figure 9:**
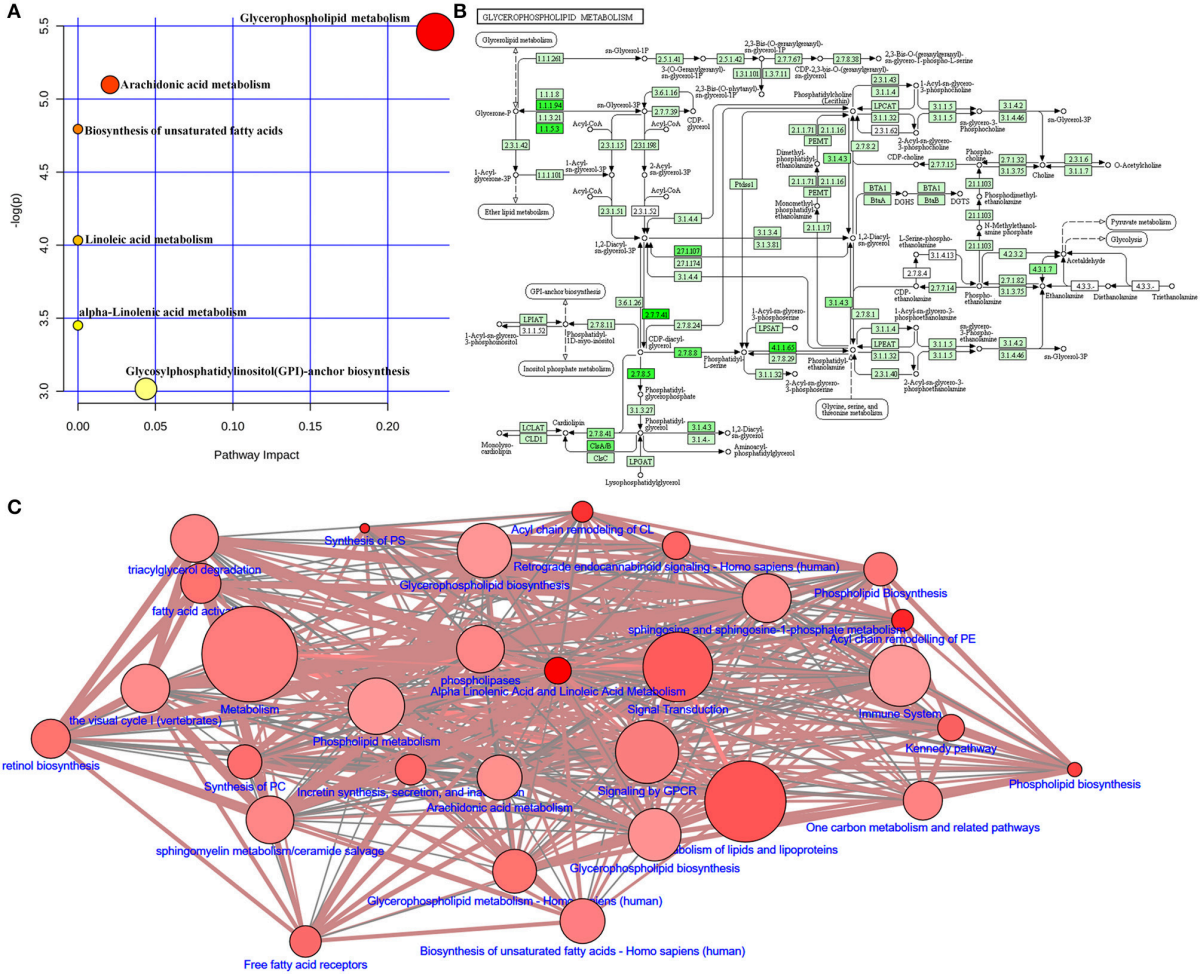
Lipid metabolic pathway analysis of identified differential lipid species. **(A)** Summary of IPA with MetPA including glycerophospholipid metabolism, arachidonic acid metabolism, biosynthesis of unsaturated fatty acids, linoleic acid metabolism, α-linolenic acid metabolism, and glycosylphosphatidylinositol (GPI)-anchor biosynthesis from significantly different lipid species. The size and color of each circle is based on pathway impact values and *p*-values, respectively. **(B)** Overview of glycerophospholipid metabolism with MetPA (reference map by KEGG). Green boxes represent enzymatic activities with putative cases of analogous activities in rats. **(C)** Lipid metabolic networks were constructed by using Wikipathways, Signalink, SMPDB, Reactome, PhosphoSitePlus, NetPath and MatrixDB. Adenine-induced CKD were associated with glycerophospholipid metabolism, triacylglycerol degradation, fatty acid activity, arachidonic acid metabolism and metabolism of lipids and lipoproteins.

Adenine triggers an imbalance between lipid synthesis and degradation. To understand the functional role of the altered lipids, we employed the KEGG database using Metaboanalyst. We evaluated both a test for over-representation of altered lipid species within a pathway (hypergeometric tests), and the impact of the altered lipid species on the function of the metabolic pathway via alterations in critical junction points of the metabolic pathway (relative between centrality). The results obtained by the 82 mouse pathways from the KEGG database were plotted to highlight the most significant metabolic pathways according to the hypergeometric test *p*-values (Y-axis) and impact (X-axis) (Figure 9A). The top three pathways in CKD by *p*-value (top two) or impact (top one) were identified, including glycerophospholipid metabolism, arachidonic acid metabolism, and biosynthesis of unsaturated fatty acids (Table 2). As an example, detailed information of the glycerophospholipid metabolism is represented in Figure 9B. The remaining pathways are shown in Figures S1–S4. Alteration of these pathways in CKD indicates that the disturbance of certain central lipid species has an important impact on multiple metabolic lipid pathways that are interconnected. A pathway enrichment overview of altered lipids highlights the arachidonic acid metabolism, as well as γ-linolenic acid, and linolenic acid metabolism for its remarkable enrichment in quantitative lipidomics from adenine-induced CKD rats vs. control rats (data not shown).

**Table 2:** Ingenuity pathway analysis **(A)** and sphingolipid metabolism **(B)** with MetPA from lipid metabolites^a^.

**Pathway Name**	**Match Status**	***p***	**−log(*p*)**	**Holm *p***	**FDR**	**Impact**	**Details**
Glycerophospholipid metabolism	2/30	0.0042	5.45	0.344	0.223	0.230	KEGG
Arachidonic acid metabolism	2/36	0.0061	5.09	0.488	0.223	0.020	KEGG
Biosynthesis of unsaturated fatty acids	2/42	0.0082	4.79	0.653	0.223	0.000	KEGG
Linoleic acid metabolism	1/5	0.0177	4.03	1.000	0.359	0.000	KEGG
alpha-Linolenic acid metabolism	1/9	0.0317	3.45	1.000	0.514	0.000	KEGG
Glycosylphosphatidylinositol(GPI)-anchor biosynthesis	1/14	0.0490	3.01	1.000	0.661	0.0439	KEGG

a *“Match status” is the total number of lipid metabolites in the pathway; the raw p is the original p calculated from the enrichment analysis; the Holm p is the p-value adjusted by Holm–Bonferroni method; the impact is the pathway impact value calculated from pathway topology analysis*.

To map the pathways overrepresented by the identified lipid species from adenine-induced CKD, identified lipid metabolic networks were constructed using various databases to determine the set that was most enriched by these lipid species. By analyzing known pathways, the image information outcome presented the biological pathway information associated with CKD. The pathway overrepresentation analysis of lipid species showed that glycerophospholipid metabolism, triacylglycerol degradation, fatty acid activity, phospholipid biosynthesis and metabolism, arachidonic acid metabolism, biosynthesis of unsaturated fatty acids, γ-linolenic acid and linolenic acid metabolism, and metabolism of lipids and lipoproteins were significantly overrepresented in adenine-induced CKD (Figure 9C).

## Discussion

Phytochemical studies have shown that the main class of compounds in AO is the terpenoids including sesquiterpenes, diterpenes and triterpenes. The total terpenoid content in AO was 53.1% as determined by UV-Vis analysis at 555 nm. Moreover, Alisol B was the main constituent of AO that ameliorated proteinuria, an increase in systolic blood pressure, and changes in histopathological parameters in nephritic rats. The Alisol B content was 3.61%, as determined by HPLC at a wavelength of 208 nm. Alisol acetates have been found to significantly lower TC, TG, and LDL-C levels in hyperlipidemic mice, while raising HDL-C levels (Xu et al., 2016). Moreover, AO has been used in the treatment of diabetes as a traditional folk medicine of China for decades (Lin, 2012). After 6 h administration, the volumes of excreted urine for the petroleum ether fraction, ethyl acetate fraction, n-Butanol fraction, and remaining H_2_O fraction were 1.8 ± 0.12, 6.5 ± 0.22, 2.3 ± 0.17, and 2.0 ± 0.18 ml/100 g, respectively. Thus, the ethyl acetate fraction in AO has the strongest drug activity among the four extraction fractions.

Inflammation and oxidative stress promote progressive interstitial fibrosis. In addition, glomerulosclerosis initially manifests as proteinuria and may eventually result in renal failure (Zhao et al., 2015a). Current pharmacological and metabolomics data indicate the presence of kidney inflammation in adenine-induced CKD rats. CKD rats demonstrate a significant increase in nuclear translocation of p65 (activation of NF-κB pathway), and the up-regulation of inflammatory, pro-oxidant and down-regulation of anti-oxidant proteins, and up-regulation of pro-fibrotic proteins (Tesch and Young, 2017). We found that inflammation of CKD rats was related to significant alterations in serum levels of various lipid metabolites. The LIPID MAPS consortium has defined lipids as hydrophobic or amphiprotic compounds that originate in whole or in part by carbocation-based condensations of the isoprene group, or by carbanion-based condensations of the ketoacyl group (Tian et al., 2014). Based on this definition, lipids were divided into eight categories, including fatty acids, sphingolipids, glycerolipids, glycerophospholipids, saccharolipids, prenol lipids, sterol lipids, and polyketides. In this study, 14 lipid species were chosen based on univariate or multivariate statistical analysis. The pathway overrepresentation analysis of lipid species demonstrated that, in adenine-induced CKD rats, 30 metabolic pathways were associated with identified lipid species. These included, five diglycerides, four triglycerides, three fatty acids, one phosphatidylcholine, and one phosphatidylethanolamine that were associated with nephropathy effects of AO on CKD. Our current study demonstrated that AO attenuated renal fibrosis by down-regulating inflammation, and mitigating lipid metabolism in CKD rats.

### Fatty acid metabolism

In adenine-induced CKD rats, significantly decreased levels of three polyunsaturated fatty acids, including 8,9-EET, EPA, and DHA were observed. These changes were completely restored by treatment with AO. Cytochrome P-450 epoxygenases metabolizes arachidonic acid into four regioisomeric epoxyeicosatrienoic acids (EET), including 5,6-EET, 8,9-EET, 11,12-EET, and 14,15-EET by catalyzing the epoxidation of the olefinic bonds of arachidonic acid (Kim et al., 2014). Previous studies have indicated that EET has potential anti-inflammatory, antihypertensive, profibrinolytic, and anti-fibrotic effects (Imig et al., 2002; Spector and Norris, 2007; Kaspera and Totah, 2009). Soluble epoxide hydrolase converts EET to the less active dihydroxyeicosatrienoic acid. EET that has been increased by the inhibition of soluble epoxide hydrolase exerts nephropathic effects, such as hypertensive renal damage, diabetic nephropathy, and obstructive nephropathy (Kim et al., 2015). It has been shown that 5/6 nephrectomized rats contained significantly decreased serum levels of EET (Zhang K. et al., 2015). Kidney tissues increase EET levels by preventing their degradation to inactive dihydroxyeicosatrienoic acids, which exhibit antihypertensive, cardio- and nephropathic activities. In a recent study, it was shown that serum 8,9-EET levels were significantly decreased in patients with advanced CKD (Chen H. et al., 2017).

EPA and DHA are omega-3 polyunsaturated fatty acids (ω-3 PUFA). Several clinical studies have suggested that ω-3 PUFA exhibits beneficial effects on patients with end-stage renal disease (Svensson et al., 2006; Lok et al., 2012). Moreover, it has been reported that compared with healthy individuals, patients with CKD have reduced levels of plasma n-3 PUFA (Madsen et al., 2011). Furthermore, it was demonstrated in many studies that in hemodialysis patients, ω-3 PUFA is significantly decreased (Gharekhani et al., 2014; Hung et al., 2015; Umemoto et al., 2016). Highly prevalent systemic inflammation is related to a high mortality rate due to cardiovascular issues in CKD patients who are on dialysis. In several studies it was demonstrated that in the general population, long-chain EPA and DHA are cardioprotective (De Caterina, 2011). The anti-inflammatory effects of EPA and DHA are considered one of the most relevant mechanisms associated with CKD and the beneficial effects of ω-3 PUFA (Spencer et al., 2013). In one study, it was indicated that EPA decreased the production of cytokines in lipopolysaccharide-induced cytokine production (Saifullah et al., 2007; Spencer et al., 2013). Previous studies have shown a significant decrease in the levels of EPA and DHA in kidney tissues derived from CKD rats (Zhao et al., 2013). In the present study, we demonstrated that in adenine-induced CKD rats, a significant reduction in EPA and DHA levels is associated with an increase in systemic inflammation, particularly in nuclear factor NF-κB p65, and the chemokines COX-2 and iNOS. Additionally, it has been reported that in kidney tissues of CKD rats, a significant reduction in EPA and DHA levels is associated with up-regulation of the TGF-β1 level (Zhao et al., 2013). Accumulated evidence has demonstrated that adenine-induced CKD results in a significant reduction in serum levels of 8,9-EET, EPA, and DHA that are associated with elevated levels of inflammation and renal fibrosis in adenine-induced CKD rats. Therefore, significantly increased levels of 8,9-EET, EPA, and DHA caused by AO treatment may be associated with inflammation improvement in adenine-induced CKD rats.

### Glycerolipid metabolism

Compared with control rats, significantly increased levels of TG (46:6), TG (64:9), TG (60:14), and TG (68:12) as well as significantly decreased levels of DG (44:6), DG (35:1), DG (40:9), DG (42:4), and DG (46:6) were observed in adenine-induced CKD rats. Patients with CKD demonstrated disturbed metabolisms of glycerolipids including monoglycerides, diglycerides, and triglycerides. Previous studies have indicated significant alterations in glycerolipid synthesis and catabolism in patients with CKD (Chen H. et al., 2017). Most previous studies have measured total TG levels in patients with CKD. It has been demonstrated that the triglyceride level of a low-density lipoprotein was significantly increased in CKD patients (Reis et al., 2015). However, increased levels of triglyceride were commonly accompanied by significantly reduced levels of HDL-C in CKD patients (Piperi et al., 2004). Hypertriglyceridemia in CKD was associated with impaired triglyceride clearance caused by down-regulation of lipoprotein lipase and a very low-density lipoprotein receptor (Vaziri et al., 2012). Moreover, it has been shown that a 3-month diglyceride intake lowered the level of abdominal fat and retarded serum lipid profiling in hemodialysis patients. Diglyceride intake significantly reduced serum levels of very low-density lipoproteins, altered monoglycerides, and elevated high-density lipoprotein levels at 3 months (Teramoto et al., 2004). Taken together, increased triglyceride and decreased diglyceride levels were consistent with clinical biochemical findings, as well as with previously published reports. The present study demonstrated that in the adenine-induced rats, both increased levels of triglyceride and decreased levels of diglyceride are restored by treatment with AO, indicating that AO may attenuate the perturbations of glycerolipids synthesis and catabolism.

Several studies have demonstrated that the degree of renal tubular interstitial injury positively correlates with an increase in oxidative stress and inflammation after renal injury (Djamali, 2007). Therefore, in this study we examined whether renal tubular interstitial injury was associated with increased oxidative stress. We found that renal tubular interstitial injury was associated with NF-κB activation, up-regulation of pro-oxidant and pro-inflammatory activities, and down-regulation of Nrf2 activity and its down-stream antioxidant and cytoprotective proteins. These findings were accompanied by activated canonical TGF-β1/Smad pathways (Zhao et al., 2013; Shang et al., 2016). Our results also revealed that renal tubular interstitial injury dramatically increased levels of TGF-β1, Smad2, Smad3, Smad4, and significantly down-regulated the activities of Smad7 in CKD rats. In summary, our findings demonstrated that AO treatment attenuated renal damage and that the mechanism may in part involve suppression of oxidative stress, inflammation and the TGF- β1/Smad signaling pathway.

## Conclusion

Pharmacological and UPLC-HDMS-based lipidomic approaches have been successfully developed to investigate lipid-lowering effects and tubular interstitial fibrosis of AO on adenine-induced CKD rats. Serum lipidomics revealed profound alterations in adenine-induced CKD rats, including serum levels of polyunsaturated fatty acids, diglycerides, and triglycerides. Pathway over-representation analysis showed that 30 metabolic pathways were associated with identified lipid species in adenine-induced CKD rats. These findings were associated with activation of NF-κB/Nfr2 and TGF-β/Smad, and pro-fibrotic signaling pathways. However, studies that focus on dose-response patterns of the underlying molecular mechanisms and clinical applications are still needed. In conclusion, we have identified the therapeutic effect of AO on CKD, and demonstrated a dual mechanism of action, including a lipid-lowering effect, and a tubular interstitial fibrosis effect.

## Author contributions

Y-YZ was responsible for the conception and design of the study; and FD, HM, J-WW, LC, MW, and HC for the data collection, analysis, and image processing. FD, HC, and Y-YZ wrote the manuscript; and A-DW revised it. FD and HC discussed the study concept and were responsible for the final approval of the version to be submitted. All authors read and approved the final manuscript.

### Conflict of interest statement

The authors declare that the research was conducted in the absence of any commercial or financial relationships that could be construed as a potential conflict of interest.
