# Supplementary material for: An Integrated Lipidomics and Phenotype Study Reveals Protective Effect and Biochemical Mechanism of Traditionally Used Alisma orientale Juzepzuk in Chronic Kidney Disease
Source: Front Pharmacol. 2018 Feb 8;9:53. doi: 10.3389/fphar.2018.00053 (PMC5809464; doi:10.3389/fphar.2018.00053)

Supplementary Material

An Integrated Lipidomics and Phenotype Study Reveals Protective Effect and Biochemical Mechanism of Traditionally Used *Alisma Orientale* Juzepzuk in Chronic Kidney Disease

Fang Dou[1†], Hua Miao[2†], Jing-Wen Wang[1], Lin Chen[2], Ming Wang[2], Hua Chen[2], Ai-Dong Wen[1*], Ying-Yong Zhao[2*]

[1] Department of Pharmacy, Xijing Hospital, Fourth Military Medical University, Xi'an, 710032, China

[2] Key Laboratory of Resource Biology and Biotechnology in Western China, Ministry of Education, Northwest University, No. 229 Taibai North Road, Xi’an, Shaanxi 710069, China

[†]Co-First authors.

FIGURE S5 | ROC curves of 14 lipid species. PLS-DA-based ROC curves of the 14 lipid species. The AUC, 95%CI, sensitivities and specificities are indicated.

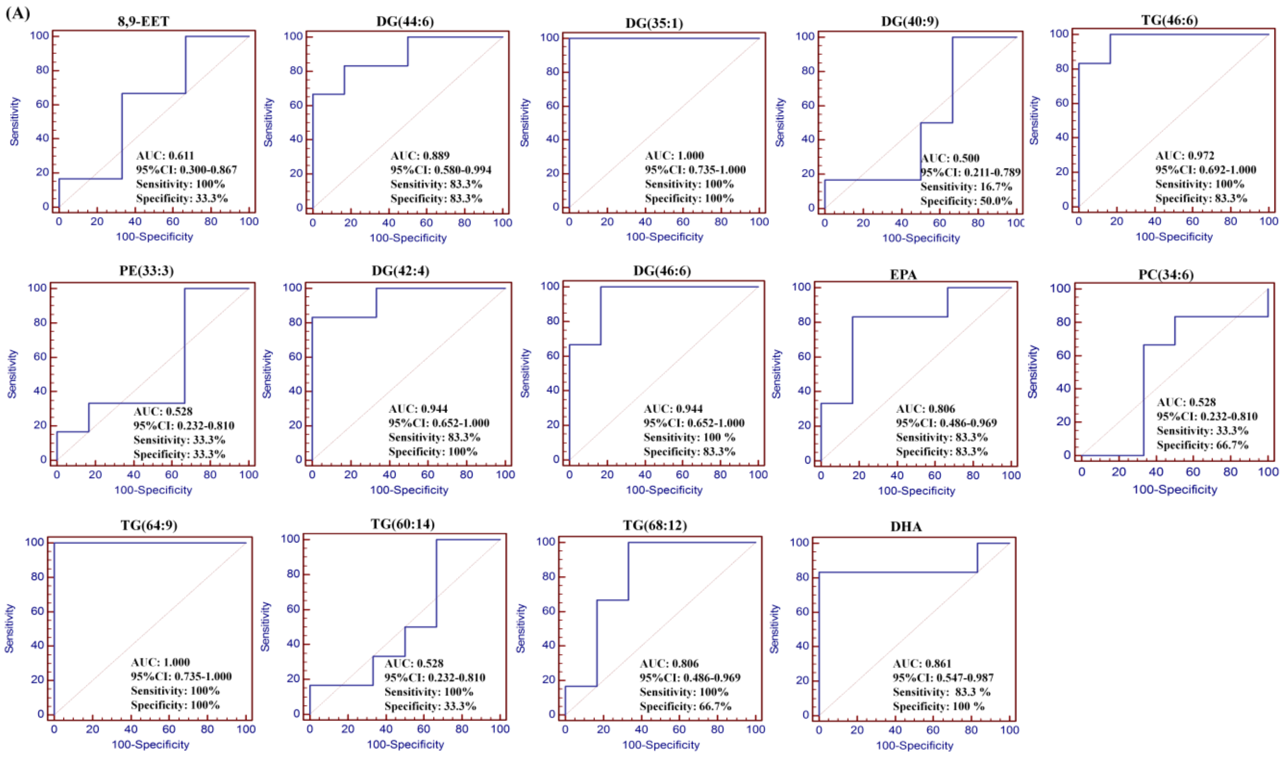

Supplement: Supplementary file 5 [file Image5.pdf]
