# Supplementary material for: An Integrated Lipidomics and Phenotype Study Reveals Protective Effect and Biochemical Mechanism of Traditionally Used Alisma orientale Juzepzuk in Chronic Kidney Disease
Source: Front Pharmacol. 2018 Feb 8;9:53. doi: 10.3389/fphar.2018.00053 (PMC5809464; doi:10.3389/fphar.2018.00053)
Supplement: Supplementary file 6 [file Table1.pdf]

Supplementary Material

An Integrated Lipidomics and Phenotype Study Reveals Protective Effect and Biochemical Mechanism of Traditionally Used *Alisma Orientale* Juzepzuk in Chronic Kidney Disease

Fang Dou[1†], Hua Miao[2†], Jing-Wen Wang[1], Lin Chen[2], Ming Wang[2], Hua Chen[2], Ai-Dong Wen[1*], Ying-Yong Zhao[2*]

[1] Department of Pharmacy, Xijing Hospital, Fourth Military Medical University, Xi'an, 710032, China

[2] Key Laboratory of Resource Biology and Biotechnology in Western China, Ministry of Education, Northwest University, No. 229 Taibai North Road, Xi'an, Shaanxi 710069, China

[†]Co-First authors.

TABLE S1 The historical sources and the uses of *Alisma orientale* Juzepzuk.

Historical sources	Traditional uses	References
Shen Nong's Herbal Classic	Promoting water metabolism	Gu, (2007)
Compendium of Materia Medica	Excreting dampness and eliminating edema	Li, (2007)
Treatise on Cold Febrile Diseases	Promoting water metabolism	Zhang, (2013)
Chinese Materia Medica	Treating edema and promoting urinary excretion	Chinese Materia Medica, (1998)
Ben Cao Yan Yi	Treating dysuria and promoting water metabolism	Kou, (1990)
Chinese Pharmacopoeia	Treating edema and urine negative	Chinese Pharmacopoeia, (2010)
