# Supplementary material for: An Integrated Lipidomics and Phenotype Study Reveals Protective Effect and Biochemical Mechanism of Traditionally Used Alisma orientale Juzepzuk in Chronic Kidney Disease
Source: Front Pharmacol. 2018 Feb 8;9:53. doi: 10.3389/fphar.2018.00053 (PMC5809464; doi:10.3389/fphar.2018.00053)
Supplement: Supplementary file 7 [file Table2.pdf]

Supplementary Material

An Integrated Lipidomics and Phenotype Study Reveals Protective Effect and Biochemical Mechanism of Traditionally Used *Alisma Orientale* Juzepzuk in Chronic Kidney Disease

Fang Dou[1†], Hua Miao[2†], Jing-Wen Wang[1], Lin Chen[2], Ming Wang[2], Hua Chen[2], Ai-Dong Wen[1*], Ying-Yong Zhao[2*]

[1] Department of Pharmacy, Xijing Hospital, Fourth Military Medical University, Xi'an, 710032, China

[2] Key Laboratory of Resource Biology and Biotechnology in Western China, Ministry of Education, Northwest University, No. 229 Taibai North Road, Xi'an, Shaanxi 710069, China

[†]Co-First authors.

TABLE S2 Triterpenes in the *Alisma orientale* Juzepzuk.

No.	Chemical name	Chemical Formula	Molecular Weight
1	Alisol B 23-acetate	$C_{31}H_{48}O_5$	500.72
2	Alisol B	$C_{29}H_{46}O_4$	458.68
3	Alisol C 23-acetate	$C_{31}H_{46}O_6$	514.70
4	Alisol C	$C_{29}H_{44}O_5$	472.67
5	11-Deoxy-alisol B	$C_{29}H_{46}O_3$	442.68
6	11-Deoxy-alisol B 23-acetate	$C_{31}H_{48}O_4$	484.72
7	11-Deoxy-alisol C	$C_{29}H_{44}O_4$	456.67
8	11-Deoxy-alisol C 23-acetate	$C_{31}H_{46}O_5$	498.70
9	16-Methyoxy-alisol 23-acetate	$C_{32}H_{50}O_5$	514.75
10	16-Hethyoxy-alisol 23-acetate	$C_{31}H_{48}O_6$	516.72
11	Alisol M 23-acetate	$C_{31}H_{46}O_7$	530.70
12	Alisol N 23-acetate	$C_{31}H_{48}O_6$	516.72
13	16β-Methoxyalisol B diacetate	$C_{34}H_{52}O_7$	572.78
14	16β-Hydroxyalisol B-triacetate	$C_{35}H_{52}O_8$	600.79
15	Alisol B diacetate	$C_{33}H_{50}O_6$	542.76
16	Alismakectone A 23-acetate	$C_{31}H_{46}O_7$	530.70
17	Alisol A	$C_{29}H_{48}O_5$	476.70
18	Alisol A	$C_{31}H_{50}O_6$	518.73
19	Alisol A 24-acetate	$C_{29}H_{46}O_6$	490.68
20	16-Oxo-alisol A	$C_{30}H_{50}O_5$	490.72
21	25-O-methoxy-alisol A	$C_{29}H_{44}O_4$	456.67
22	Alisol H	$C_{29}H_{46}O_4$	458.68
23	11-deoxyalisol A	$C_{29}H_{46}O_5$	474.68
24	23-O-Methylalisol A	$C_{30}H_{50}O_5$	490.72
25	11,23,25-Tri-O-alisol A	$C_{33}H_{58}O_5$	534.82
26	Alisol A 23,24-diacetate	$C_{33}H_{52}O_7$	560.77
27	Alisol E	$C_{29}H_{48}O_5$	476.70

28	Alisol E 23-acetate	$C_{31}H_{50}O_6$	518.73
29	Alisol E 24-acetate	$C_{31}H_{50}O_6$	518.73
30	13β,17β-epoxy-alisolB	$C_{30}H_{48}O_5$	488.71
31	Alisol D	$C_{32}H_{50}O_6$	530.75
32	11-Deoxy-13,17-epoxy-alisol B 23-acetate	$C_{32}H_{50}O_5$	514.75
33	11-deoxyalisol D	$C_{30}H_{48}O_4$	472.71
34	Alisol D acetate	$C_{34}H_{52}O_7$	572.78
35	Alisol L 23-acetate	$C_{32}H_{46}O_5$	510.71
36	13,17-Epoxy-alisol A	$C_{30}H_{50}O_6$	506.72
37	11-Deoxy-13,17-epoxy-alisol A	$C_{30}H_{50}O_5$	490.72
38	13,17-Epoxy-alisol A 24-acetate	$C_{32}H_{52}O_7$	548.76
39	16,23-Oxido-alisol B	$C_{31}H_{48}O_4$	484.72
40	Alisol I	$C_{31}H_{48}O_3$	468.72
41	Alisol K 23-acetate	$C_{32}H_{46}O_6$	526.71
42	Alismalactone 23-acetate	$C_{36}H_{52}O_8$	612.80
43	Neoalisol	$C_{30}H_{48}O_5$	488.71
44	Neoalisol 11,24-diacetate	$C_{34}H_{52}O_7$	572.78
45	Alisol F	$C_{30}H_{48}O_5$	488.71
46	Alisol F 24-acetate	$C_{32}H_{50}O_6$	530.75
47	Alisol F diacetate	$C_{34}H_{52}O_7$	572.78
48	25-Anhydro-alisol A(Alisol G)	$C_{30}H_{48}O_4$	472.71
49	25-Anhydro-alisol A 11-acetate	$C_{32}H_{50}O_4$	498.75
50	25-Dehydroxy-alisol A 24-acetate	$C_{32}H_{50}O_4$	498.75
51	Alisol J 23-acetate	$C_{31}H_{44}O_6$	512.69
52	24-deacetyl alisol O	$C_{30}H_{46}O_4$	470.69
53	alisol O	$C_{32}H_{48}O_5$	512.73
54	11,25-anhydro-alisol F	$C_{30}H_{44}O_3$	452.68
